# Assessment of serum cobalamin (vitamin B12) status among Human Immunodeficiency Virus (HIV) infected Nigerians with anaemia using serum methylmalonic acid

**DOI:** 10.4314/ahs.v25i4.5

**Published:** 2025-12

**Authors:** Ngozi Ugwu, Omolade Awodu, Collins Ugwu, Godwin Bazuaye

**Affiliations:** 1 Federal Teaching Hospital Abakaliki, Haematology; 2 Department of Haematology, University of Benin, Benin City, Nigeria; 3 Ebonyi State University Faculty of Clinical Medicine, Internal Medicine; 4 Department of Haematology, Igbinedion University Teaching Hospital, Okada, Edo State, Nigeria

**Keywords:** Anaemia, Cobalamin, Human Immunodeficiency virus, methylmalonic acid

## Abstract

**Background:**

Cobalamin deficiency has been implicated as a contributor to anaemia in HIV disease. This study aimed to determine cobalamin status among antiretroviral therapy (ART)-naive HIV-infected patients with anaemia.

**Methods:**

A cross-sectional study was conducted and two categories of participants were recruited: ART-naive HIV-positive patients with anaemia (Group A) and HIV-negative individuals with anaemia (Group B). Socio-demographic and medical history was obtained. Methylmalonic acid (MMA) levels (an indirect measure of cobalamin level) was carried out by ELISA method. Full blood count was determined using haematology autoanalyser. CD4 count were done only on HIV-infected subjects using cyflowmeter. Data was analysed with SPSS software, version 20.

**Results:**

A total of 180 participants comprising 126 group A and 54 group B, aged between 18 and 60 years were studied. Cobalamin deficiency (indicated by high MMA level) was found to be significantly higher among group A participants (p=0.001). There was negative correlation between haemoglobin level and MMA (r= -0.147) (p= 0.547), as well as between CD4 count and MMA (r= -0.327) (p= 0.171), though not statistically significant. The age group commonly infected by HIV was 31 to 40years (42.1%). More females were affected with HIV disease with male to female ratio of 1:3.5.

**Conclusion:**

HIV-infection is associated with cobalamin deficiency and so contribute to anaemia of HIV infection. Larger population studies are recommended to ascertain the veracity of these findings.

## Introduction

Human immunodeficiency virus (HIV) is a lentivirus of the retrovirus family which is the aetiologic agent of Acquired Immunodeficiency Syndrome (AIDS). The first cases of AIDS was discovered among homosexual men in the United States in 1981^1^. The men manifested with unusual type of pneumonia called pneumocystic carinii pneumonia and rare skin tumours called Kaposi's sarcoma. The disease was subsequently recognised in Africa and Western Europe but has now become a global pandemic. HIV infects about 0.6% of the world's population^2^. The cells of the immune system such as helper T cell (CD4+ T cells), dendritic cells and macrophages are affected with consequent reduction in the level of CD4+ T cells. Depletion of CD4+ T cells decline below a critical level leads to loss of cell-mediated immunity and the affected individual becomes progressively susceptible to opportunistic infections^3^.

Clinical consequences of HIV infection include a host of infections and malignancies that rarely cause illnesses in immunocompetent individuals. Clinical manifestation varies according to sex, age, geographic location, race, treatment status and lifestyle^4^. Virus replication and worsening of immunological status continues throughout the course of the disease in infected individuals. Patients therefore manifest with diseases involving different body systems including hematopoietic organ/tissue.

Haematological abnormalities are among the most common complications of advanced HIV infection and include qualitative and quantitative marrow defects, immune cytopenias, effects of opportunistic infections, and a myriad of drugs against infections or malignancy^5^. It is therefore not surprising that peripheral blood and bone marrow abnormalities are common in HIV infection with such a varied assault on the haematopoietic system.

Anaemia has been reported to be the most common haematological manifestation of HIV infection^6^. Anaemia is an independent risk factor for death, and recovery from anaemia is associated with improved prognosis. A number of factors contribute to anaemia in HIV infection, including cobalamin deficiency. Reduced cobalamin level has been reported in 10-35% of patients infected with HIV^6^. A stage of negative cobalamin balance has been suggested in HIV infection as the serum transcobalamin II cobalamin-binding capacity is elevated even in asymptomatic patients^6^. CD4 cell count tend to fall along with cobalamin level as HIV disease progresses to AIDS, hence decreasing cobalamin levels are predictors of disease progression^7^.

Assessment of cobalamin status is conventionally based on the measurement of serum cobalamin levels. However, it has been reported that about 50% of patients with subclinical disease have normal cobalamin levels^8^. Cobalamin deficiency leads to an increase in serum methylmalonyl-CoA and its metabolic product, methylmalonic acid (MMA) (measured as an indicator of cobalamin status)^9^. Varying degrees of cobalamin status among HIV infected patients have been reported by studies done in different parts of the world^10^,^11^, but little is known of cobalamin status among HIV-infected individuals in our locality. The aim of this study was to determine cobalamin status among antiretroviral therapy (ART)-naive HIV-infected patients with anaemia.

## Materials and methods

### Study design and study area

The study was a cross-sectional study conducted at the University of Benin Teaching Hospital (UBTH), Benin City, Nigeria.

### Study population

Study population were adults with HIV infection and anaemia (Group A) and HIV-negative patients with anaemia (group B) aged between 18 and 60years.

Anaemia in this study was defined as haemoglobin level less than 12mg/dl for females and less than 13mg/dl for males, according to WHO haemoglobin threshold for anemia^12^.

### Sample size determination

Using the Fisher's formula equation^13^ for cross sectional study and a prevalence of cobalamin deficiency of 9% from previous study^14^, a sample size of 126 was calculated. However, 180 participants were studied (126 HIV positive subjects with anaemia and 54 HIV-negative subjects with anaemia).

### Inclusion criteria

For Group A: established HIV- infected subjects with anaemia. Group B -individuals without HIV infection but anaemic, all subjects aged 18 to 60years.

### Exclusion criteria

The following subjects were excluded from the study: pregnant women and nursing mothers, persons who have had surgery involving the stomach or ileum, vegetarians, patients with other comorbidities such as diabetes mellitus, renal failure, malignancies such as leukaemia, polycythaemia vera; those on drug therapy known to cause B12 deficiency (e.g., phenytoin, neomycin, metformin, omeprazole) and those on cobalamin supplementation.

### Sampling technique

Participants for this study (Group A – HIV-infected patients with anaemia) were recruited consecutively from HIV clinic of the University of Benin Teaching Hospital while group B (HIV-negative anaemic individuals) were recruited consecutively from general out-patient department of the hospital.

HIV infection was diagnosed in blood samples using rapid diagnostic kit (Determine HIV1/2 and Unigold™). Where there is a disparity between the two kits, samples were sent for confirmatory tests by Enzyme-linked immunosorbent assay.

### Data collection and sample analysis

The tool for data collection was a pre-tested, semi-structured, interviewer-administered questionnaire. Information sought in the questionnaire included socio-demographic, drug history, dietary history, medical and surgical history. Five millilitres of venous blood sample was collected aseptically via antecubital vein from all the participants after proper cleaning of the venepuncture site with cotton wool soaked with methylated spirit. About 3mls of blood was dispensed into Ethylene Diamine Tetraacetic acid (EDTA) bottle and used for determination of full blood count using haematology autoanalyser (manufactured by Erma Inc Tokyo, model PCE-210).

Blood in EDTA bottles were also used to determine CD4 only on HIV-positive patients with anaemia using cyflometer (Cyflow partec) (made in Germany with model number 050217517).

The remaining blood sample was dispensed into plain bottle, allowed to cloth and the serum separated and used for analysis of serum methylmalonic acid (MMA) level (an indirect measure of cobalamin level) by ELISA method using microplate washer and microplate reader (made in USA by Diagnostic Automation Inc, with model number DAR 800) and human methylmalonic acid ELISA kit (made by Wuhan EIAab Science Co. Ltd, China). Serum MMA was used as an indirect measure of serum cobalamin level because MMA level has been reported as a better indicator of cobalamin deficiency than cobalamin level itself^15^. In cobalamin deficiency, MMA accumulates in the blood and so it is used as an indirect measure of cobalamin status in this study.

## Ethical issues

The study was approved by the Research and Ethics Committee of University of the Hospital, with registration number: ADM/E22/A/VOL.VII/606. Informed written consent was obtained from each participant before being included in the study.

### Data analysis

Data collected from this study were analysed using the statistical package for the social science (SPSS) software, version 20 (Chicago, IL, USA). Descriptive statistics was used to compute percentages and proportions, means and standard deviation. Frequencies of non-parametric variables were compared using Chi square test. Unpaired student T-test was used to compare means of parametric variables and Pearson's correlation test was used to determine the relationship between parametric variables. P-values less than 0.05 was considered significant.

## Results

A total of 180 subjects participated in this study, and were composed of 126 ART-naive HIV-positive patients with anaemia (group A), 54 subjects who were HIV-negative subjects with anaemia (group B). The participants were aged between 18years and 60years. Group A had a mean age of 33 ±6.7 years while group B had mean age of 33.5 ±10.8years. The age and sex distribution of the participants is shown in Table 1.

**Table 1:** Socio-demographic profile of the participants

Parameters	Group A (HIV plus anaemia) n(%)	Group B (no HIV but anaemic) n(%)	Total n (%)	P-value
**Gender**				
Male	28 (22.2)	11 (20.4)	39 (21.7)	0.782
Female	98 (77.8)	43 (79.6)	141 (78.3)	
Total	126 (100)	54 (100)	180 (100)	
**Age**				
18 - 30	49 (38.9)	17 (31.5)	66 (36.7)	
31 - 40	53 (42.1)	26 (48.1)	79 (43.9)	
41 - 50	18 (14.3)	9 (16.7)	27 (15.0)	
51 - 60	6 (4.7)	2 (3.7)	8 (4.4)	
Total	126 (100)	54 (100)	180 (100)	

Chi square (X2) = 0.076; p-value = 0.782, n = number

Among group A participants, there were 28 (22.2%) males and 98 (77.8%) females with male to female ratio of 1:3.5. The age group with the highest frequency of HIV infection was 31–40 years (42.1). This was followed by 18–30-year age group (38.9%) (Table 1).

### Blood count of the participants

Full blood count done showed that the average white blood cell (WBC) count of the group A was 4.6 X 10^9^/l ±0.2 whereas that of group B was 4.7 X109/l ±1.1. Mean WBC count between the two groups was not statistically significant (P= 0.252). The mean haemoglobin level of group A was 9.0±1.5g/dl while that of group B was 10.5 ±0.8g/dl, though not statistically significant (P = 0.237). The mean lymphocyte count among group A was 1.9 ±0.7 X 10^9^/l whereas that of group B was 1.9 ±0.6 X 109/l. but the difference was not statistically significant (P= 0.788). The mean platelet count between the two groups were also not statistically significant (P = 0.435) (Table 2).

**Table 2:** complete blood count of the participants

Blood count	Group A (anaemia with HIV)	Group B (anaemia without HIV)	P-value
Haemoglobin level (g/dl)	9.0±1.5	10.5±0.8	0.237
White blood cell count (x 10^9^/l)	4.6±0.2	4.7±1.1	0.252
Lymphocyte count (X 10/l)	1.9±0.7	1.9±0.6	0.788
Platelet count (X 10^9^/l)	239±87	260±77	0.435

### Serum MMA levels of the subjects

**Figure 1:**
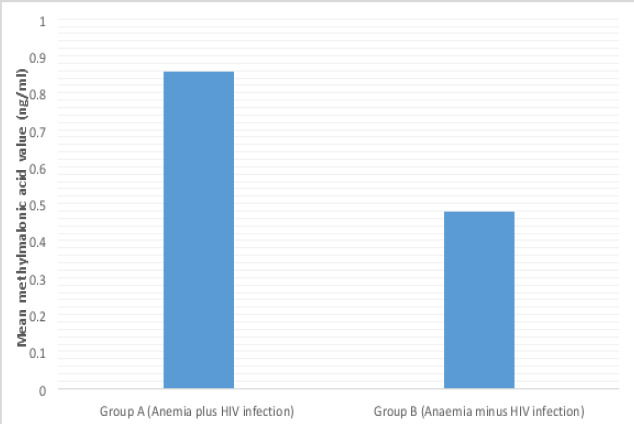
Mean methylamalonic acid level in subjects with anaemia and HIV infection (group A) and those with anaemia without HIV infection (groupB)

The mean serum MMA level of group A (HIV positive with anaemia) was 0.86ng/ml ±0.4 whereas that of Group B (HIV negative with anaemia) was 0.48 ±0.1ng/ml. The mean difference in serum MMA level between the two groups was statistically significant (P= 0.001) (Figure 1).

### Correlation between serum MMA level with haemoglobin level and CD4 count among HIV positive anaemic patients

**Figure 2:**
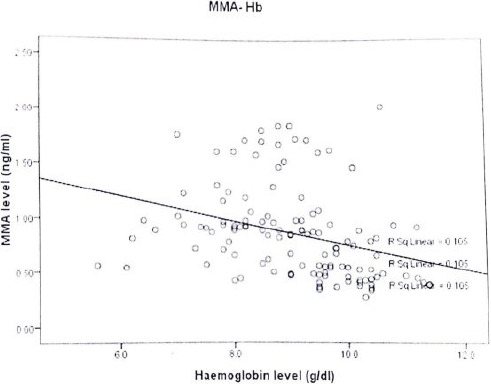
Scatter diagram showing the relationship between methylmalonic acid and haemoglobin level

**Figure 3:**
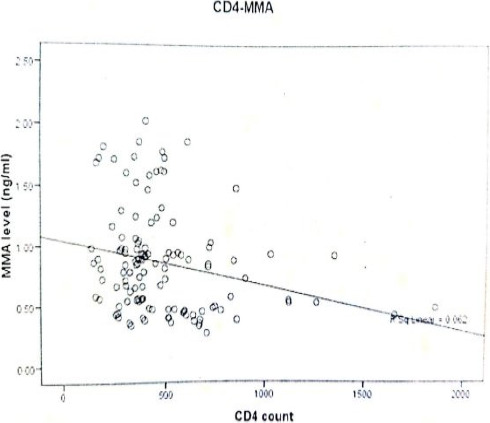
Scatter diagram showing the relationship between methylmalonic acid level and CD4 count

Correlation of Serum MMA level with haemoglobin level among ART-naive HIV-infected subjects with anaemia showed an inverse relationship (r= -0.147) (p= 0.547), though not significant (Figure 2). Similarly, correlation of serum MMA and CD4 count among ART-naive HIV-infected subjects with anaemia showed an inverse correlation, though not statistically significant (r = -317) (p= 0.185) (Figure 3).

## Discussion

Anaemia is a common haematological manifestation of HIV-infection and has been reported to be associated with HIV disease progression and decreased survival^16^. Studies have reported high prevalence of anaemia among HIV-infected patients^6^,^16^. Causes of anaemia in HIV infection is multifactorial, ranging from the effect of the virus itself, other concurrent opportunistic infections, effect of drugs used in treatment, malignancies associated with HIV infection such as non-Hodgkins lymphoma and Kaposi sarcoma, malnutrition with vitamin deficiencies including cobalamin deficiency^17^.

This study assessed cobalamin status using serum MMA (which is a metabolite of cobalamin) in HIV-positive adults. Different screening assays for B12 deficiency are available, but this study measured serum MMA level as an indicator of vitamin B12 status. This is because serum MMA has been found to be a more sensitive and earlier marker of cobalamin deficiency than serum cobalamin^15^. This study found that serum MMA was significantly higher (an indication of low cobalamin status) among HIV positive subjects with anaemia (Group A) compared to HIV negative subjects with anaemia (group B). This is in agreement with previous studies by Kalejaiye et al.^10^ in Lagos; Semeere et al.^18^ in Uganda; Adhikari et al.^11^ in India and Hepburn et al.^19^ in USA. Low cobalamin status (indicated by high MMA level) found in this study and other studies may be due to decreased absorption of cobalamin as a result of HIV enteropathy^20^. Evidence has shown that there is villous atrophy and gastric mucosal damage in HIV-infected patients with reduced ability to secrete enzymes and acids which may cause difficulty in splitting cobalamin from food and so makes it unavailable for absorption^20^. It has also reported that cobalamin depletion was found in patients infected with HIV who were asymptomatic leading to the possibility that cobalamin depletion could serve as an early marker of HIV infection^18^. These findings suggest strongly that HIV-infection is associated with cobalamin deficiency.

This study also revealed an inverse relationship between CD4 count and serum MMA level, thus CD4 count falls as serum MMA level rises, though not statistically significant. This is in keeping with the study conducted by Kavitha et al.^7^ who reported that low cobalamin is associated with falling CD4 count. Similarly, Semeere, et al, reported that HIV patients with sub-optimal cobalamin level had a higher mean rate of CD4 decline compared to those with normal cobalamin level^18^. Low CD4 count seen in HIV infection is due to progressive destruction of CD4 cells by the virus^21^.

Increasing MMA level was also found to be associated with progressive fall in haemoglobin level, though not statistically significant. Previous studies have also reported low haemoglobin level and anaemia with associated elevated MMA in patients infected with HIV^7^,^22^. High MMA level associated with anaemia in HIV-infected patients may be attributed to villous atrophy and gastric mucosal damage reported in patients with HIV infection, with reduced ability to secrete enzymes and acids making it difficult to split cobalamin from food^23^. Thus cobalamin unavailable for absorption with resulyanat cobalamin deficiency and ultimately anaemia. Cobalamin deficiency will also inhibit conversion of methylmalonic acid to succinic acid leading to accumulation of MMA in the blood and consequently raised serum MMA level. Low haemoglobin level and associated with low cobalamin level found in this study is not surprising considering the fact that cobalamin deficiency inhibits purine and thymidylate syntheses, impairs DNA synthesis, and causes erythroblast apoptosis, with resultant anemia from ineffective erythropoiesis^24^.

This study also found that young adults were commonly affected by HIV infection as the most commonly affected age group was 31 to 40 years (42.1%), followed by 18 to 30years (38.9%), both contributing over three quarter of HIV infected individuals. This is consistent with findings of previous studies which also reported HIV infection to be commoner among young people^25^,^26^. This is attributed to the fact that HIV-infection has been noted to be common among sexually active age group who are young adults that constituted the bulk of the work force and may predict an unfavourable effect on the socioeconomic situation of the country. Thus anaemia as a result of cobalamin deficiency may constitute serious morbidity that keep patients away from work and is a major cause of mortality in HIV-infected patients^27^.

This study showed that there were more HIV-infected females than males with male to female ratio of 1: 3.5. This is in keeping with findings of previous studies which reported that more females are affected by HIV infection than males^25^,^26^. Certain factors has been found to contribute to the higher prevalence of HIV infection among females. These include unhealthy cultural practices in our environment, non empowerment of majority of females economically compared to males^25^ and also because they go for medical check more than males and may be detected in the course of investigation for other reasons. For instance, many of them were detected during their routine ante-natal visit^28^.

## Limitation of the study

Renal function test was not done to rule out renal impairment as a cause of elevated serum methylmalonic acid level.

## Conclusion

Cobalamin deficiency (measured by serum MMA level) is commonly found in HIV infection and so contribute significantly to anaemia of HIV infection. Cobalamin deficiency is more common in HIV-infected patients with anaemia compared to those without HIV infection. Larger population studies are recommended to ascertain the veracity of these findings.
